# Characterization of Community Acquired *Staphylococcus aureus* Associated with Skin and Soft Tissue Infection in Beijing: High Prevalence of PVL^+^ ST398

**DOI:** 10.1371/journal.pone.0038577

**Published:** 2012-06-06

**Authors:** Chunjiang Zhao, Yingmei Liu, Mingze Zhao, Yali Liu, Yong Yu, Hongbin Chen, Qiuning Sun, Huawei Chen, Wei Jiang, Yudong Liu, Shaomei Han, Yingchun Xu, Minjun Chen, Bin Cao, Hui Wang

**Affiliations:** 1 Department of Clinical Laboratory, Peking University People’s Hospital, Peking University, Beijing, China; 2 Peking Union Medical College Hospital, Chinese Academy of Medical Sciences, Beijing, China; 3 Department of Infectious Diseases and Clinical Microbiology, Beijing Chao-Yang Hospital, Capital Medical University, Beijing, China; 4 Department of Clinical Laboratory, Beijing Pinggu Hospital, Beijing, China; 5 Department of Clinical Laboratory, The First Hospital Affiliated to the People’s Liberation Army General Hospital, Beijing, China; 6 Graduate Biomedical Sciences, University of Alabama at Birmingham, Birmingham, Alabama, United States of America; National Institutes of Health, United States of America

## Abstract

Adult community-associated methicillin-resistant *Staphylococcus aureus* (CA-MRSA) and methicillin-susceptible *S aureus* (CA-MSSA) skin and soft tissue infection (SSTI) in China is not well described. A prospective cohort of adults with SSTI was established between January 2009 and August 2010 at 4 hospitals in Beijing. Susceptibility testing and molecular typing, including multilocus sequence typing, *spa, agr* typing, and toxin detection were assessed for all *S. aureus* isolates. Overall, 501 SSTI patients were enrolled. Cutaneous abscess (40.7%) was the most common infection, followed by impetigo (6.8%) and cellulitis (4.8%). *S. aureus* accounted for 32.7% (164/501) of SSTIs. Five isolates (5/164, 3.0%) were CA-MRSA. The most dominant ST in CA-MSSA was ST398 (17.6%). The prevalence of Panton-Valentine Leukocidin (*pvl*) gene was 41.5% (66/159) in MSSA. Female, younger patients and infections requiring incision or drainage were more commonly associated with *pvl*-positive *S. aureus* (P<0.03); *sec* gene was more often identified in CC5 (P<0.03); *seh* gene was more prevalent in CC1 (P = 0.001). Importantly, ST59 isolates showed more resistance to erythromycin, clindamycin and tetracycline, and needed more surgical intervention. In conclusion, CA-MRSA infections were rare among adult SSTI patients in Beijing. Six major MSSA clones were identified and associated with unique antimicrobial susceptibility, toxin profiles, and *agr* types. A high prevalence of livestock ST398 clone (17.1% of all *S. aureus* infections) was found with no apparent association to animal contact.

## Introduction

Methicillin resistant *Staphylococcus aureus* (MRSA) infection represents a significant cause of morbidity and mortality in both hospital and community settings. Community-associated MRSA (CA-MRSA) has become increasingly important as a cause of skin and soft tissue infections (SSTIs), particularly in patients presenting to emergency departments [1]–[3]. In China, HA-MRSA has been extensively studied during the past years with ST239-SCC*mec* III as the predominant clone [4], [5]. However, data regarding CA-MRSA was limited, with mainly reporting from children. According to these studies, the prevalence of CA-MRSA from SSTI among children was 4%, and ST59-MRSA-IV-t437 was the most common clone among CA-MRSA isolates [6]. To our knowledge, no prospective clinical studies of SSTIs from adults caused by CA-MRSA have been reported in China.

The origin of the staphylococcal cassette chromosome mec (*SCCmec*) in CA-MRSA is still unknown. It is hypothesized that MRSA probably originated through the transfer of SCCmec into extant MSSA lineages with a genetic background common to MRSA clones. Therefore, it is very important to investigate the clonal structure of MSSA in each country or region, and to compare with MRSA. Moreover, as the virulence factors play an important role in the pathogenesis of community-acquired *S. aureus* infections, we also investigated the prevalence of virulence factors, including Panton-Valentine Leukocidin (*pvl*), *staphylococcal* enterotoxins C (*sec*) and *staphylococcal* enterotoxins H (*seh*) in these *S. aureus* isolates and their relationship with the genetic background.

In this study, we prospectively enrolled 501 SSTI patients from 2 teaching hospitals and 2 community hospitals in Beijing in order to investigate the prevalence, clinical and molecular characteristics of SSTIs in adults caused by *S. aureus*, with emphasis on the prevalence of CA-MRSA in adult SSTIs in China. To our surprise, we found that the livestock-associated clone ST398 in MSSA was the most prevalent with unique antimicrobial susceptibility, toxin profiles, and *agr* types.

## Materials and Methods

### Enrollment of Patients and Isolation of Bacterial Isolates

From January 2009 to August 2010, consecutive outpatients with SSTIs were prospectively enrolled at the surgical clinic and dermatological clinics in two teaching hospitals (Peking Union Medical College Hospital and Beijing Chao-Yang Hospital) and two community hospitals (Beijing Pinggu Hospital and The First Hospital Affiliated to the People’s Liberation Army General Hospital). The annual visits of surgical clinic and dermatological clinics of these four hospitals ranged from 4000 to 6,000. Community acquisition of *S. aureus* was defined as a positive culture from outpatients, or inpatients within 48 hours admission if they had no risk factors for healthcare-associated acquisition such as recent hospitalization, surgery, hemodialysis, the presence of any permanent in-dwelling catheter or percutaneous medical device, or residence in a long-term care facility. A Case Report Form (CRF) completed for each patient that included demographic information, clinical symptoms, laboratory findings, the type of infection diagnosed, all antibiotic use, and clinical outcome. Specimens were collected from infection sites of every patient enrolled and cultured on blood agar and Eosin methylene blue agar. According to the colony morphology and Gram stain, rapid methods were used for *S. aureus* identification [7]. MRSA isolates were initially identified using cefoxitin and oxacillin disks (30 µg, Oxoid, Cambridge) and confirmed for the presence of the *mec*A gene by polymerase chain reaction (PCR) as described previously [8].

Institutional Review Board approval was obtained before this study was begun. Written informed consent was obtained from all patients at the time of enrollment.

### Antimicrobial Susceptibility Testing

Antimicrobial susceptibility profiles of *S. aureus* isolates were determined by the agar dilution method on Mueller-Hinton agar, according to the recommendations and definitions from Clinical and Laboratory Standards Institute (CLSI) [9]. Antimicrobial agents tested included oxacillin (Sigma Chemical, St. Louis, MO), cefoxitin (Sigma Chemical, St. Louis, MO), teicoplanin (,Sanofi-aventis, Schiltigheim, France), vancomycin (Eli Lilly, Indianapolis, Indiana, United States), cefazolin (Sigma Chemical, St. Louis, MO), cefuroxime (Sigma Chemical, St. Louis, MO), ceftriaxone (Sigma Chemical, St. Louis, MO), erythromycin (Sigma Chemical, St. Louis, MO), clindamycin (Sigma Chemical, St. Louis, MO), tetracycline (Sigma Chemical, St. Louis, MO), chloramphenicol (Sigma Chemical, St. Louis, MO), gentamicin (Sigma Chemical, St. Louis, MO), trimethropim/sulphamethoxazole (SXT, Sigma Chemical, St. Louis, MO), rifampin (Sigma Chemical, St. Louis, MO), levofloxacin (Daiichi Pharmaceutical, Tokyo, Japan), tigecycline (Pfizer Pharmaceuticals, New York, USA (formerly Wyeth Pharmaceuticals, Collegeville, PA.), linezolid (Pfizer), and mupirocin (Sigma Chemical, St. Louis, MO). ATCC29213 (*S. aureus*) and ATCC29212 (*Enterococcus faecalis*) were used as quality controls.

### Molecular Typing Methods

All of the *S. aureus* isolates were investigated by multilocus sequence typing (MLST), *spa* typing, and accessory gene regulator (*agr*) typing. Suspensions of overnight *S. aureus* cultures on blood agar were lysed by lysostaphin for phenol-chloroform extraction of the genomic DNA, which was reconstituted in 1 ml Tris-EDTA buffer for PCR reactions. MLST was carried out as described previously [10] comparing sequences of the PCR products to the MLST website (http://saureus.mlst.net) with clustering of related STs into clonal complexes (CCs) utilizing the online eBURST program [11]. Purified PCR products of *spa* were sequenced and repeat patterns and *spa* types were assigned from the *spa* database [12]. SCC*mec* typing was performed on the CA-MRSA isolates using the multiplex PCR protocol of Oliveira et al [13]. Nontypeable (NT) types were those differing from the standard types. International clones of SCC*mec* types I to V were used as quality controls. *agr* typing was performed as previously described method [14].

### Detection of Toxin Genes

All of the *S. aureus* isolates were screened for Panton-Valentine Leukocidin (*pvl*) gene, Toxic shock syndrome toxin 1(*tsst-1*) gene, *Staphylococcal* enterotoxin C (*sec*), *Staphylococcal* enterotoxin H (*seh*), and *Staphylococcal* exfoliative toxin (*et*) gene by PCR and electrophoresis with primers as previously described [15].

### Statistical Analysis

The χ^2^ test with Yastes’s correction or Fisher’s exact test using SPSS, version 13.0 (SPSS, Chicago, IL, USA) were used for analyzing the quantitative variables. A P value of ≤0.05 was considered statistically significant. All susceptibility data and molecular test results were analyzed using WHONET software, version 5.6.

## Results

### Clinical and Microbiological Characteristics of SSTIs

From January 2009 to August 2010, a total of 501 SSTI cases were enrolled in this study. Demographic details, clinical and laboratory features, and antibiotic usage are shown in Table 1. Of all 501 cases enrolled, *S. aureus* was the most frequent organism, accounting for 32.7% (164 isolates) of all SSTIs.

**Table 1:** Demographic and clinical features of patients with skin and soft-tissue infections (n = 501).

Variables	n(%)
Gender-male	319 (62.3)
Age (mean±SD, years)	41.4±18.8
range	18–92
Co-morbidities	
Cardiovascular disease	42 (8.4)
Diabetes mellitus	35 (6.9)
Respiratory disease	13 (2.6)
Mean white blood cells (range,10^9^/L) n = 145	8.59 (4.1–26.9)
White blood cells >10×10^9^/L	26.9%
Neutrophils (mean±SD,%) n = 123	72.7±11.0
Hemoglobin (mean±SD, g/L) n = 140	13.8±2.7
Platelet (mean±SD,10^9^/L) n = 136	236±80
Duration of symptoms, median, Quartile, (days)	
Fever (T>37°C), n = 50	3 (1–5)
Redness, n = 157	6 (4–10)
Swelling, n = 154	6 (4–10)
Warmth, n = 119	5 (4–8)
Pain, n = 144	5 (3–8)
Pathogen identified	
*Single pathogen*	
MSSA	159 (31.7)
MRSA	5 (1.0)
Coagulase negative Staphylococcus (CoNS)	101 (20.2)
*Streptococcus spp*	24 (4.8)
*Escherichia coli*	21 (4.2)
*Klebsiella spp*	17 (3.4)
*Proteus spp*	8 (1.6)
Others	66 (13.2)
Multiple pathogens	25 (5.0)
Types of skin and soft-tissue infection	
Cutaneous abscess	204 (40.7)
Impetigo	34 (6.8)
Traumatic wound infection	30 (6.0)
Cellulitis	24 (4.8)
Panaritium	24 (4.8)
Furuncles, and carbuncles	20 (4.0)
Mastitis	13 (2.6)
Lymphangitis	12 (2.4)
Diabetic foot infection	11 (2.2)
Folliculitis	10 (2.0)
Pyomyositis	10 (2.0)
Others	108 (23.5)
Antibiotic use only	153 (30.6)
Drainage or surgical intervention only	20 (4)
Antibiotic with surgical intervention	328 (65.4)
Admission to hospital	57 (14.9)
Admission to ICU	2 (0.6)
Length of hospital stay (mean±SD, days)	16.9±14.5
Death	0

### Resistance Profile of *S. aureus* Isolates

Of all the 164 *S. aureus,* 159 isolates were MSSA, and 5 isolates were confirmed as MRSA. All MSSA isolates were susceptible to most antibiotics tested, including cefazolin (susceptible rate, 100%), cefuroxime (100%), ceftriaxone (100%), trimethoprim/sulfamethoxazole (98.7%), levofloxacin (97.5%), and chloramphenicol (95%). However, the susceptibility of MSSA to erythromycin, clindamycin, gentamicin and tetracycline was lower with the susceptibility rates of 41.5%, 71.7%, 79.9%, and 81.0%, respectively. Additionally, 22.6% (36/159) MSSA isolates were resistant to more than 3 antimicrobial classes simultaneously. In contrast, all 5 CA-MRSA isolates were only susceptible to vancomycin, teicoplanin, linezolid, daptomycin, and tigecycline. No high level resistance to mupirocin was observed in either MSSA or CA-MRSA isolates (MIC range, 0.016–64 μg/ml).

### Clinical and Molecular Characteristics of *S. aureus*

Among the *S. aureus* infections, cutaneous abscess was the most common infection, followed by cellulitis, and impetigo. All of the *S. aureus* were analyzed by MLST, *spa* and *agr* typing. A total of 30 STs and 56 *spa* types were identified in 164 *S. aureus*. Clustering analysis by eBURST showed that these STs belonged to 16 clonal complexes (CCs). Six clones (ST398, CC7, CC1, CC5, ST59, CC8) were found to be predominant types, constituting 17.1% (28/164), 12.2% (20/164), 11.6% (19/164), 8.5% (14/164), 6.7% (11/164) and 6.1% (10/164), respectively.

Overall, *agr* type I was identified as the predominant type, accounting for 64.6% (106/164) of all *S.aureus* isolates, followed by type III (14.0%), type II (13.4%), and type IV (7.9%). No significant difference on the prevalence of *pvl* was found among four *agr* types. Interestingly, none of the isolates with *agr* IV carried *sec* gene, while 59.1%–60.9% of the isolates with *agr* type II and III produced SEC (*P*<0.001). *agr* III had higher prevalence of s*eh* gene than others (39.1% vs. 2.8%–7.7%, *P*<0.001). In addition, the isolates with *agr* III and IV carried more *tsst*-1 genes (15.4%–17.4%) than those with *agr* I (1.9%, P = 0.005).

When evaluating only MSSA isolates, the most dominant ST was ST398 (17.6%), followed by ST7 (11.9%), ST1 (6.9%), ST59 (6.3%), ST5 (5.7%), and ST6 (5.0%). The most common spa types in MSSA were t034 (12/159, 7.5%), t796 (10/159, 6.3%), t571 (9/159, 5.7%), t127 (9/159, 5.7%), and t189 (9/159, 5.7%). The molecular characteristics for the MSSA strains are listed in Table 2.

**Table 2:** Molecular characteristics of 159 methicillin-susceptible *S. aureus* isolates.

ST	MLST-CC	No. (%) of Isolates	No. of *pvl*-positive (%)	No. of *sec-*positive (%)	No. of *seh*-positive (%)	No. of *tsst*-1-positive (%)	*agr* types (No. of isolates)	*spa* types (No.of isolates)
398	398	28 (17.6)	18 (64.3)	3	3	0	I (26), II (1), IV (1)	t034(12), t571(9), t011(4), t588(1), t4387(1), t3625(1)
7	7	19 (11.9)	0	4	0	0	I (19)	t796(10), t091(6), t803(1), t309(1), t2740(1)
943	7	1 (0.6)	0	0	0	0	I (1)	t091(1)
59	59	10 (6.3)	8	6	1	0	I (9), II (1)	t437(5), t441(2), t163(2), t4362(1)
1	1	11 (6.9)	0	8	8	0	III (11)	t127(8), t045(1), t286(1), t701(1)
188	1	8 (5.0)	0	1	0	1	I (8)	t189(8)
5	5	9 (5.7)	2	8	0	1	II (9)	t002(5), t2164(2), t062(1), t045(1)
965	5	3 (1.9)	2	3	1	0	II (1), III (2)	t062(1), t127(1), t164(1)
72	5	2 (1.3)	1	1	1	0	I (1), II (1)	t148(1), t179(1)
6	6	8 (5.0)	1	1	0	0	I (8)	t701(7), t1944(1)
25	25	7 (4.4)	5	5	0	0	I (7)	t078(3), t349(1), t280(1), t258(1), t1460(1)
30	30	7 (4.4)	7	5	0	4	I (1), II (2), III (4)	t318(5), t964(1), t6952(1)
285	30	1 (0.6)	1	1	0	1	III (1)	t318(1)
1320	30	1 (0.6)	0	0	0	1	III (1)	t363(1)
15	15	6 (3.8)	0	1	0	0	II (4), IV(2)	t084(4), t5864(1), t2119(1)
199	15	1 (0.6)	0	0	0	0	II (1)	t368(1)
22	22	6 (3.8)	6	1	0	0	I (6)	t309(5), t3622(1)
88	88	5 (3.1)	5	0	0	0	II (2), III(3)	t796(1), t6497(1), t4431(1), t2616(1), t2592(1)
121	121	4 (2.4)	2	0	0	0	IV (4)	t159(2), t7290(1), t1425(1)
95	121	3 (1.9)	2	0	0	2	I (1), IV (2)	t164(1), t645(1), t7292(1)
1301	121	1 (0.6)	1	0	0	0	IV (1)	t3666(1)
800	121	1 (0.6)	0	0	0	0	IV (1)	t1425(1)
20	20	2 (1.2)	2	2	0	0	I (2)	t164(1), t881(1)
45	45	2 (1.2)	0	2	0	0	I (2)	t015(1), t1460(1)
630	8	7 (4.4)	0	0	0	0	I (7)	t377(5), t441(1), t7291(1)
Other	Singleton	6 (3.8)	3	0	0	0	I (4), III (1), IV (1)	t269(1), t4431(1), t5338(1), t701(1), t015(1), t189(1)
Total		159	66	51	14	10	I (102), II (22), III (23), IV (12)	

The 5 patients with CA-MRSA infections occurred during work or daily activities without any identifiable risk factors. Two of them were farmers, who developed infection after trauma during work. The third was a female worker who had mastitis, the fourth was a retired woman who developed spontaneous abscesses on her check, and the last one was a 25-year old exchange student from Turkey with a spontaneous abscess on the abdomen. All of the five patients were treated with antibiotics and surgical drainage. Notably, although the antibiotics were later shown to be inactive against MRSA, all 5 patients cured 5 to 7 days after surgical intervention. The clinical and molecular characteristics of 5 CA-MRSA isolates are shown in Table 3.

**Table 3:** Clinical and molecular features of 5 cases with CA-MRSA infections.

Case No.	Age (yr)	Gender	Residence	Infection	Resistance Profile^a^	SCC*mec* b	ST	MLST-CC	*spa* types	*agr* types	Toxins^c^		
											SEC	SEH	PVL
1	72	Female	China	Cutaneous abscess	OEH	NT	ST6	CC6	t701	I	–	–	–
2	25	Male	Turkey	Cutaneous abscess	OEL	IV	ST8	CC8	t008	IV	–	–	+
3	38	Female	China	Cellulitis	OENH	V	ST59	CC59	t437	I	+	–	+
4	68	Male	China	Wound infection	OENRGL	III	ST239	CC8	t632	I	+	+	–
5	26	Male	China	Wound infection	OENRGL	I	ST239	CC8	t037	I	+	–	–

a O: oxacillin, E: erythromycin, N: clindamycin, R: tetracycline, H: chloramphenicol, G: gentamicin, L: levofloxacin.

b NT: nontypable.

c All of the 5 isolates were negative for TSST-1 toxin.

### Comparison of Clinical Characteristics between *pvl*-positive and *pvl*-negative *S. aureus* SSTIs

Overall, 66 of 159 MSSA (41.5%) and 2 of 5 CA-MRSA (40.0%) isolates harbored the *pvl* gene. Female patients were found more commonly in *pvl*-positive group than in *pvl*-negative group (47.1% vs. 28.7%, P = 0.017), and the mean age among *pvl*-positive patients was younger than *pvl*-negative group (37.8±16.4 vs. 45.5±21.0, P = 0.014). In addition, the percentage of patients who needed surgical intervention was significantly higher in *pvl*-producing *S. aureus* SSTI than that in *pvl*-negative group (60.7% vs. 42.0%, *p* = 0.032). However, no significance was found between two groups with underlying diseases, types of infections, severity of illness and clinical outcomes, such as symptoms alleviation time and length of stay in hospital,

### Comparison of Clinical and Molecular Features among the Major MLST Clones

Comparison of clinical features, toxins and *agr* types was performed among the six major clones (Table 4). More patients in ST59 group needed incision or drainage (*p*<0.001). Moreover, significant difference was found on antimicrobial susceptibility to erythromycin, clindamycin, tetracycline and gentamicin among the 6 CC clones (P values, 0.006–0.039, Table 4). ST59 was more resistant to erythromycin, clindamycin and tetracycline, while CC5 was more resistant to gentamicin.

**Table 4:** Comparison of clinical information, toxins, *agr* types and susceptibility among the major *S. aureus* clones (%)^a^.

Variables	ST398 (n = 28)	CC7 (n = 20)	CC1 (n = 19)	CC5 (n = 14)	ST59 (n = 11)	CC8 (n = 10)	*P* value
Incision or drainage	12/26	9/18	13/19	1/12	10/10	3/8	**0.001** b
Antimicrobial susceptibility							
Erythromycin	7	5	12	5	1	3	**0.039**
Clindamycin	18	12	16	6	2	8	**0.006**
Tetracycline	25	14	17	12	5	7	**0.023**
Gentamicin	21	10	17	6	9	7	**0.025**
Toxins							
*pvl* ^+^	18 (64.3)	0	0	5 (35.7)	9 (81.8)	1 (10.0)	**<0.001** c
*sec* ^+^	3 (10.7)	4 (20.0)	9 (47.4)	12 (85.7)	6 (54.5)	2 (20.0)	**<0.001** d
*seh* ^+^	3 (10.7)	0	8 (42.1)	2 (14.3)	1 (9.1)	1 (10.0)	**<0.002** e
*agr* types							**<0.001** f
I	26 (92.9)	20 (100.0)	8 (42.1)	1 (7.1)	10 (90.9)	9 (90.0)	
II	1 (3.6)	0	0	11 (78.6)	1 (9.1)	0	
III	0	0	11 (57.9)	2 (14.3)	0	0	
IV	1 (3.6)	0	0	0	0	1 (10.0)	

a Non-significant difference was found on gender, age, fever at diagnosis, contact with animals among 6 major clones.

b Significant difference was found in the following comparisons: ST59 vs. CC5 (p<0.001); ST59 vs. CC8 (p = 0.007); ST59 vs. ST398 (p = 0.003); ST59 vs. CC7 (p = 0.010); CC1 vs. CC5 (P = 0.002);

c Significant difference was found in the following comparisons: ST59 vs. CC1 (p<0.001); ST59 vs. CC5 (p = 0.042); ST59 vs. CC7 (p<0.001); ST59 vs. CC8 (p = 0.002); CC1 vs. CC5 (P = 0.008); CC5 vs. CC7 (P = 0.007); ST398 vs. CC1 (P<0.001); ST398 vs. CC7 (P<0.001); ST398 vs. CC8 (P = 0.008).

d Significant difference was found in the following comparisons: CC1 vs. CC5 (p = 0.033); CC1 vs. ST398 (p = 0.071); CC5 vs. CC7 (p = 0.000); CC5 vs. CC8 (P = 0.003); CC5 vs. ST398 (P<0.001); ST59 vs. ST398 (P = 0.008).

e Significant difference was found in the following comparisons: CC1 vs. ST398 (p = 0.018); CC1 vs. CC7 (p = 0.001).

f Significant difference was found in the following comparisons: CC1 vs.CC5 (p = 0.015); CC5 vs. either of ST398, CC7, ST59 and CC8 (p<0.001).

The prevalence of toxin genes varied among 6 clones. *pvl* gene was significantly more commonly detected in ST59 (81.8%, 9/11), ST398 (64.3%, 18/28) and CC5 (35.7%, 5/14) than in CC1(0/19) or CC7 (0/20) (*p*<0.016, *p*<0.009). In contrast, the prevalence of *sec* gene was significantly higher in CC5 (85.7%) than in CC1 (47.4%), CC7 (20%), CC8 (20%), and ST398 (10%) (P<0.005).

Interestingly, the major six clones differed in *agr* types. *agr* type I was predominant in CC7 isolates (100%), ST398 isolates (92.9%), ST59 isolates (90.9%), and CC8 isolates (90.0%). In contrast, type II was found more prevalent in CC5 isolates (78.6%), In CC1, type III and type I accounted for 57.9% and 42.1%, respectively.

## Discussion

In this study, we have three novel findings: 1) the prevalence of CA-MRSA was low among adults with SSTIs; 2) six major MSSA clones (ST398, ST7, ST1, ST59, and ST5) were responsible for SSTIs in adults in Beijing, China; 3) ST398 was the most prevalent clone among MSSA isolates.

Our previous study showed that the major HA-MSSAs belonged to the same clones (ST398, ST7, ST1, ST59, and ST5) suggesting a similar epidemiology for both hospital acquired infections and community acquired infections [4]. We confirmed the presence of ST398 clone in the community [6], [16]. Similar findings came from European researchers who found the percentage of ST398 in MRSA increased from 0 in 2002 to 30% in 2007 in Netherlands and from 13% in 2005 to 22.4% in 2008 in Germany [17], [18], suggesting both regional variation as well as evolution in *S. aureus* ST398 over time.

ST398 is usually associated with livestock infection in pigs and people exposed to animals [19], however, we did not find any association between livestock contact and ST398 infection.Whole genome sequencing of European ST398 has revealed that it lacked virulence factors such as enterotoxins and phage-encoded toxins [20]. Our findings are novel because we find a great number of strains (64.3%) harbored *pvl* gene.

*S. aureus* strains with *pvl* are associated with abscess formation and tissue necrosis. Our study indicated that the prevalence of *pvl* in CA-MSSA infection was high (41.5%), which was different from the previous study on children where only 4.2% of strains carried *pvl* [6]. The difference in the distribution of major clones between adults and children may explain this difference in *pvl* prevalence between two studies. Similarly, past investigation has found the presence of *pvl* to be associated with a enhanced inflammatory response and localized infections [21], which is in concurrent with our findings that more *pvl^+^* patients needed incision or drainage. Another study from New York also found the presence of PVL as a significant predictor for incision and drainage for MSSA infection, and they found patients infected with *pvl* ^+^ *S. aureus* were significantly younger than those infected with *pvl* ^−^ *S. aureus* [22], similar to our study.

The *agr* locus belongs to the core variable genome and is thus linked to CCs. Our molecular testing is in agreement with the findings of other studies. For example, we observed that *agr-*I was the most common type and linked to ST22, ST45, ST7, ST188, and ST59; *agr*-II was present in CC5 and CC15; *agr*-III was associated with ST1 and CC30; and *agr-4* was detected in CC121 [23], [24].

In summary, this study provides the baseline information of the epidemiology and molecular characteristics of MSSA and CA-MRSA in adults with SSTIs in Beijing – currently most disease is caused by MSSA. We identified 6 major MSSA clones that were responsible for SSTIs in the community with ST398 the most prevalent clone. Though the prevalence of CA-MRSA in Beijing is low at present, the possibility that CA-MRSA could be imported from other countries, or the potential that MSSA may acquire the SCC*mec* elements makes it important for continuous surveillance of *S. aureus* infections.
